# Supplementary material for: Dissecting the regulatory roles of ORM proteins in the sphingolipid pathway of plants
Source: PLoS Comput Biol. 2021 Jan 28;17(1):e1008284. doi: 10.1371/journal.pcbi.1008284 (PMC7872301; doi:10.1371/journal.pcbi.1008284)
Supplement: S2 File — (DOCX) [file pcbi.1008284.s002.docx]

A range of ∆G’ values (∆G’_LB_ - ∆G’_UB_) can be calculated from the standard Gibbs free energy value (∆G’^0^) and the physiological range of metabolite concentration (X_min_ = 10 $\mu$mol and X_max_ = 1 mmol) using the following equation:

$$\Delta G^{'}= \Delta G^{'0}+RTln\left( \frac{p}{s} \right)$$

Where p is calculated by multiplying product concentrations and s is calculated by multiplying substrate concentration for the reaction going in the forward direction. Therefore, for a reaction

A + B $\leftrightarrow$ C + D

The range of ∆G’ values can be calculated as follows

$$\Delta G_{LB}^{'}=\Delta G^{'0}+RTln\left( \frac{\left[ C \right]_{max}\left[ D \right]_{max}}{\left[ A \right]_{min}\left[ B \right]_{min}} \right)$$

$$\Delta G_{UB}^{'}=\Delta G^{'0}+RTln\left( \frac{\left[ C \right]_{min}\left[ D \right]_{min}}{\left[ A \right]_{max}\left[ B \right]_{max}} \right)$$

The table in the following page shows the range of Gibbs free energy values for the reactions in the sphingolipid pathway.

| **Reaction** | **∆G'_LB_ (kJ/mol)** | **∆G'_UB_ (kJ/mol)** |
| --- | --- | --- |
| SPT | -35.96811173 | 11.23688898 |
| KSR | -57.87290902 | 11.23688898 |
| SBH | -700.1780941 | -14.20094013 |
| LCBKa | -46.56720719 | -14.20094013 |
| LCBKb | -46.56720719 | -17.73397195 |
| CS1a | -72.0050363 | -17.73397195 |
| CS1b | -72.0050363 | -643.7872106 |
| CS2a | -75.53806812 | -643.7872106 |
| CS2b | -75.53806812 | -643.7872106 |
| SLDa | -701.5913068 | -643.7872106 |
| SLDb | -701.5913068 | -6.428270122 |
| SLDc | -701.5913068 | -6.428270122 |
| SLDd | -701.5913068 | -6.428270122 |
| FA2Ha | -64.2323663 | -6.428270122 |
| FA2Hb | -64.2323663 | -6.428270122 |
| FA2Hc | -64.2323663 | -6.428270122 |
| FA2Hd | -64.2323663 | -6.428270122 |
| FA2He | -64.2323663 | -6.428270122 |
| FA2Hf | -64.2323663 | -9.254695579 |
| FA2Hg | -64.2323663 | -9.254695579 |
| FA2Hh | -64.2323663 | -9.254695579 |
| GCSa | -67.05879175 | -9.254695579 |
| GCSb | -67.05879175 | -9.254695579 |
| GCSc | -67.05879175 | -9.254695579 |
| GCSd | -67.05879175 | -9.254695579 |
| GCSe | -67.05879175 | -9.254695579 |
| GCSf | -67.05879175 | -6.428270122 |
| GCSg | -67.05879175 | -6.428270122 |
| GCSh | -67.05879175 | -6.428270122 |
| GIPCSa | -64.2323663 | -6.428270122 |
| GIPCSb | -64.2323663 | -6.428270122 |
| GIPCSc | -64.2323663 | -6.428270122 |
| GIPCSd | -64.2323663 | -6.428270122 |
| GIPCSe | -64.2323663 | -6.428270122 |
| GIPCSf | -64.2323663 | -6.428270122 |
| GIPCSg | -64.2323663 | -6.428270122 |
| GIPCSh | -64.2323663 | 28.19544172 |
| DPL1a | -64.2323663 | 18.56142569 |
| DPL1b | -64.2323663 | 18.56142569 |
