# Supplementary material for: Dissecting the regulatory roles of ORM proteins in the sphingolipid pathway of plants
Source: PLoS Comput Biol. 2021 Jan 28;17(1):e1008284. doi: 10.1371/journal.pcbi.1008284 (PMC7872301; doi:10.1371/journal.pcbi.1008284)
Supplement: S3 File — (DOCX) [file pcbi.1008284.s003.docx]

Regulatory networks are incorporated into the model through an m*x*n stoichiometric matrix (S_reg_) where m is the number of metabolites in the network and n is the number of enzyme catalyzed reactions. Each position in the matrix can have one of three values:

$S_{reg}\left( m,n \right)=0$ (component m does not regulate enzyme n)

$S_{reg}\left( m,n \right)=-3$ (component m represses enzyme n)

$S_{reg}\left( m,n \right)=-4$ (component m activates enzyme n)

To enable network metabolites to regulate ORM expression, an ORM activation reaction was added

$$ORM \to ORM_{act}$$

Where only the active form of ORM (ORM_act_) can participate in regulatory interactions with other enzymes.

To consider the effect that different functional groups or fatty acid lengths had on ceramide regulation, ceramides where broken down into 7 categories as follows:

Cer I: all ceramides; Cer II: “C-16 containing” ceramides; Cer III: “VLCFA containing” ceramides; Cer IV: non-hydroxylated ceramides; Cer V: hydroxylated ceramides; Cer VI: saturated ceramides; Cer VII: non-saturated ceramides.

The table below shows the list of metabolite-enzyme interactions considered in this work

| Enzyme | Regulator |
| --- | --- |
| SPT | ORM |
|  | Cer I-VII |
| CS I | ORM |
| CS II | ORM |
| ORM activation | Cer I-VII |

The set of regulatory networks tested comprises different combinations of the interactions in the table above. When testing ceramide-enzyme interactions, the Cer I-enzyme interaction would be tested first, and if no models passed the applied filtration steps, then no further ceramide categories would likely pass as they are all considered subset of category I. Furthermore, since the two classes of CS act on different length ceramides, category II and III ceramide inhibition makes more biological sense which is why those schemes were tested as well. Finally, for the regulatory scheme which did pass all filtration steps (scheme #6) and for the regulatory schemes exclusively testing ceramide inhibition of ORM activation, a scheme was proposed for each ceramide category. The table in the following page shows a list of the 23 regulatory schemes tested and the number of kinetic parameters associated with each network.

| Scheme # | Regulated Enzyme(s) | Regulator(s) | Regulatory role | # of parameters |
| --- | --- | --- | --- | --- |
| 0 | SPT | ORM | Inhibitor | 294 |
| 1 | CS I | ORM | Inhibitor | 302 |
| 2 | CS II | ORM | Activator | 310 |
| 3 | CS I | ORM | Inhibitor | 318 |
|  | CS II | ORM | Activator |  |
| 4 | ORM activation | Cer (cat. I) | Inhibitor | 358 |
| 5 | CS I | ORM | Inhibitor | 366 |
|  | ORM activation | Cer (cat. I) | Inhibitor |  |
| 6 | CS II | ORM | Activator | 374 |
|  | ORM activation | Cer (cat. I) | Inhibitor |  |
| 7 | CS I | ORM | Inhibitor | 382 |
|  | CS II | ORM | Activator |  |
|  | ORM activation | Cer (cat. I) | Inhibitor |  |
| 8 | CS I | ORM | Inhibitor | 334 |
|  | ORM activation | Cer (cat. II) | Inhibitor |  |
| 9 | CS I | ORM | Inhibitor | 334 |
|  | ORM activation | Cer (cat. III) | Inhibitor |  |
| 10 | CS I | ORM | Inhibitor | 350 |
|  | CS II | ORM | Activator |  |
|  | ORM activation | Cer (cat. II) | Inhibitor |  |
| 11 | CS I | ORM | Inhibitor | 350 |
|  | CS II | ORM | Activator |  |
|  | ORM activation | Cer (cat. III) | Inhibitor |  |
| 12 | ORM activation | Cer (cat. II) | Inhibitor | 326 |
| 13 | ORM activation | Cer (cat. III) | Inhibitor | 326 |
| 14 | ORM activation | Cer (cat. IV) | Inhibitor | 326 |
| 15 | ORM activation | Cer (cat. V) | Inhibitor | 326 |
| 16 | ORM activation | Cer (cat. VI) | Inhibitor | 326 |
| 17 | ORM activation | Cer (cat. VII) | Inhibitor | 326 |
| 18 | CS II | ORM | Activator | 342 |
|  | ORM activation | Cer (cat. II) | Inhibitor |  |
| 19 | CS II | ORM | Activator | 342 |
|  | ORM activation | Cer (cat. III) | Inhibitor |  |
| 20 | CS II | ORM | Activator | 342 |
|  | ORM activation | Cer (cat. IV) | Inhibitor |  |
| 21 | CS II | ORM | Activator | 342 |
|  | ORM activation | Cer (cat. V) | Inhibitor |  |
| 22 | CS II | ORM | Activator | 342 |
|  | ORM activation |  | Inhibitor |  |
| 23 | CS II | ORM | Activator | 342 |
|  | ORM activation |  | Inhibitor |  |

* ORM inhibition of SPT (scheme #0) was incorporated into all subsequent schemes as this regulation is known to occur.

Determining kinetic parameters associated with regulatory reactions

The elementary steps for a unimolecular enzymatic reaction inhibited by the regulator I can be written as:

*v_i,7_*

*v_i,10_*

*v_i,9_*

*EI*

*v_i,8_*

*X_1_EI*

*v_i,3_*

*v_i,1_*

*v_i,6_*

*v_i,2_*

*v_i,4_*

*v_i,5_*

$$X_{1}+E \leftrightarrow X_{1}E \leftrightarrow X_{2}E \leftrightarrow X_{2}+E$$

Similarly, a unimolecular enzymatic reaction activated by the regulator A can be decomposed as follows:

*v_i,13_*

*v_i,11_*

*X_2_ + EA*

*X_2_EA*

*X_1_EA*

*v_i,9_*

*EA*

*v_i,14_*

*v_i,8_*

*v_i,12_*

*v_i,7_*

*v_i,10_*

*v_i,6_*

*v_i,2_*

*v_i,4_*

*v_i,5_*

*v_i,3_*

*v_i,1_*

$$X_{1}+E \leftrightarrow X_{1}E \leftrightarrow X_{2}E \leftrightarrow X_{2}+E$$

Kinetic parameters associated with regulatory elementary steps (drawn in red for inhibitory steps and green for activation steps) are sampled in a similar manner to that of kinetic parameters associated with metabolic elementary steps (detailed in the Methods section). They differ only due to the fact that the elementary reaction rates of these steps are not constrained by V_i,net_, the overall flux which determines the difference between forward and reverse rates. Therefore, the reversibility of these steps are set to 1 in the reference steady state.

$${R_{reg}}_{i,j}^{ref}=\frac{v_{i,2j-1}}{v_{i,2j}}=1$$

It is noted that these parameters are sampled during the same step in which parameters associated with metabolic steps are sampled, and not in a subsequent step.
