# Supplementary material for: Dissecting the regulatory roles of ORM proteins in the sphingolipid pathway of plants
Source: PLoS Comput Biol. 2021 Jan 28;17(1):e1008284. doi: 10.1371/journal.pcbi.1008284 (PMC7872301; doi:10.1371/journal.pcbi.1008284)
Supplement: S4 File — (DOCX) [file pcbi.1008284.s004.docx]

Calculating apparent kinetic parameters

A unimolecular elementary reaction with kinetic parameters k_1_-k_6_ is written as:

*v_i,4_*

*v_i,3_*

*v_i,1_*

*v_i,2_*

*v_i,5_*

*v_i,6_*

$$S+E \leftrightarrow ES \leftrightarrow EP \leftrightarrow P+E$$

Each elementary flux v_i_ has an associated elementary kinetic parameter. Using Briggs-Haldane kinetics (assuming $\frac{d\left[ ES \right]}{dt}=\frac{d\left[ EP \right]}{dt}=0$) [1], and solving for S and P yields the following expression:

$$v=E\frac{k_{cat}^{+}\frac{S}{K_{S}}-k_{cat}^{-}\frac{P}{K_{P}}}{1+\frac{S}{K_{S}}+\frac{P}{K_{P}}}$$

Apparent enzyme parameters $k_{cat}^{+},k_{cat}^{-}, K_{s},\mathrm{and} K_{p}$ are derived from the elementary mass-action kinetics [1] as follows:

$$k_{cat}^{+}=\frac{k_{3}k_{5}}{k_{3}+k_{4}+k_{5}}$$

$$k_{cat}^{-}=\frac{k_{2}k_{4}}{{k_{2}+k}_{3}+k_{4}}$$

$$K_{S}=\frac{k_{2}k_{4}+k_{2}k_{5}+k_{3}k_{5}}{k_{1}(k_{3}+k_{4}+k_{5})}$$

$$K_{P}=\frac{k_{2}k_{4}+k_{2}k_{5}+k_{3}k_{5}}{k_{6}({k_{2}+k}_{3}+k_{4})}$$

Where $k_{cat}$values refer to the maximal forward and backward rates per unit of enzyme, and $K_{s} \mathrm{and} K_{p}$ refer to substrate and product Michaelis constants, respectively [2].

Two sample Kolmogorov-Smirnov (KS) test

The KS test [3] is commonly used to determine whether two samples come from the same distribution. After calculating the lumped kinetic parameters for the ensemble of models sampled for the regulatory scheme displayed in Fig 4 of the manuscript, two samples were created for each lumped parameter. The first sample contained parameters that passed all filtration models (8 data points for each parameter) and the second sample comprised parameters that did not pass (99 data points for each parameters). The MATLAB function kstest2 was used with a cutoff value of 0.001. The output was a vector indicating which of the parameters had a significantly different distribution between the two samples.

**References**

1. Haldane JBS. The course of enzymatic reactions and its mathematical theory. In: Enzymes. Longmans, Green and Co. London; 1930. p. 74–92.

2. Noor E, Flamholz A, Liebermeister W, Bar-Even A, Milo R. A note on the kinetics of enzyme action: A decomposition that highlights thermodynamic effects. FEBS Lett [Internet]. 2013 Sep 2;587(17):2772–7. Available from: https://doi.org/10.1016/j.febslet.2013.07.028

3. Massey FJ. The Kolmogorov-Smirnov Test for Goodness of Fit. J Am Stat Assoc [Internet]. 1951 Mar 1;46(253):68–78. Available from: https://www.tandfonline.com/doi/abs/10.1080/01621459.1951.10500769
