# Supplementary material for: Dissecting the regulatory roles of ORM proteins in the sphingolipid pathway of plants
Source: PLoS Comput Biol. 2021 Jan 28;17(1):e1008284. doi: 10.1371/journal.pcbi.1008284 (PMC7872301; doi:10.1371/journal.pcbi.1008284)
Supplement: Attachment — Submitted filename: Response to Reviewers.docx [file pcbi.1008284.s005.docx]

We would like to thank the reviewers for the time and effort put in to reviewing this manuscript. Below are our point by point responses to the comments raised. Additions made in the revised manuscript to respond to the reviewers’ comments have been highlighted in red. Other edits have been tracked and submitted as a ‘Related Manuscript’ file.

**Specific responses**

Reviewer #1:

1) It would be useful to provide a (supplementary) figure with a growth curve, comparing experimentally determined and simulated growth rates.

Response:

We thank the reviewer for providing this recommendation. The growth curve and experimentally determined growth rate have been added to S1 file (line 510-511). Furthermore, since the experimental growth rate was set as a constraint when generating the reference steady-state, both experimental and simulated growth rates are the same.

2) Please distribute code (e.g., Matlab scripts, Github link) and make all necessary data (e.g., sbml models) available in order to be able to reproduce the results.

Response:

All scripts used in this work have been uploaded to a github repository, and the link has been provided in the manuscript (line 532).

3) In S1 file, could include GPR associations of the 24 genes linked to the 78 reactions.

Response:

GPR associations using both gene symbols and AGI codes have been added.

4) Please enumerate the 23 postulated schemes used to predict the regulation of the sphingolipid pathway. S3 file does not provide sufficient information for each scheme.

Response:

An additional table was added to supplementary file 3 enumerating all 23 schemes.

5) As the authors mentioned in the discussion, this study lacks experimental validation of the model-generated secondary regulatory mechanism of sphingolipids (“ceramide-ORM-ceramide synthase (class II) regulatory interaction”). If possible, it would be good to include some experimental validation.

Response:

For experimental validation of the proposed regulatory mechanism (ceramides-ORMs-CSII), we are in the process of conducting co-immunoprecipitation (co-IP) experiments to determine the physical interaction of ORM1/2 proteins with ceramide synthases (LOH1/2/3). In addition, we will conduct *in vitro* assays to measure the activity of ceramide synthases using microsomal protein isolated from Arabidopsis mutants and transgenic lines and yeast expressing the Arabidopsis ORMs/LOHs. we will also evaluate the changes in activity associated with feeding different concentrations of short chain ceramides.

Although these experiments will likely provide results that are informative for our modeling efforts, they are not trivial and require significant time and effort for the generation of the materials and the optimization of the methods. Therefore, it will be very difficult to include these results as experimental validation for this paper. Specifically, for the co-IP experiments, a set of DNA constructs (to express the proteins of study with different molecular tags) need to be designed, constructed and validated before performing the experiments.

6) What are some limitations of using this approach?

Response:

As described in the discussion (line 430-440), one of the main limitations is the inability to test all possible regulatory mechanisms due to computational intractability. This means that other regulatory schemes could potentially satisfy the applied filtration steps, which is why we emphasize that these predictions constitute the first step in a continuously updated cycle aimed at minimizing the need for experimental testing. Another limitation which may have some implication on the predictions is associated with the Ensemble Modeling framework's assumption that the relative change in enzyme concentration between the reference state and perturbed state is negligible for non-perturbed enzymes. Although outside the scope of the present study, proteomic analysis could potentially be carried out on each of the perturbed cell lines to determine the validity of this assumption.

7) Please include references to previously published genome-scale metabolic models of Arabidopsis and highlight the advantage of using a pruned sphingolipid biosynthetic pathway in this study.

Response:

We have added a new paragraph to the introduction highlighting the role that genome-scale metabolic models have played in advancing our understanding of plant metabolism (especially that of Arabidopsis) and have referenced such models. We also highlighted how such models aim to answer a different set of questions related mainly to primary metabolism and metabolic shifts under different conditions. As the reviewer has pointed out, such models are not equipped to account for enzyme and metabolite concentrations and are therefore unable to capture regulatory interactions.

We also deeply thank Reviewer #1 for all the minor edits brought to our attention. We have attempted to rectify all the points brought up to the best of our ability:

1) What is the evidence that this hypothetical regulatory mechanism is shared across different plants? I advise the authors to use a more specific term in the title.

Response:

Based on phylogenetic analysis of pathway enzymes as well as the plant sphingolipid literature, we believe that the hypothesized regulatory mechanism would be generalizable to the plant kingdom due to the observed conservation of ORMs, ceramide synthases and other enzymes of the sphingolipid pathway across plants (1–3).

**References**

1.	Luttgeharm KD, Kimberlin AN, Cahoon EB. Plant Sphingolipid Metabolism and Function BT - Lipids in Plant and Algae Development. In: Nakamura Y, Li-Beisson Y, editors. Cham: Springer International Publishing; 2016. p. 249–86. Available from: https://doi.org/10.1007/978-3-319-25979-6_11

2.	Chueasiri C, Chunthong K, Pitnjam K, Chakhonkaen S, Sangarwut N, Sangsawang K, et al. Rice ORMDL controls sphingolipid homeostasis affecting fertility resulting from abnormal pollen development. PLoS One [Internet]. 2014 Sep 5;9(9):e106386–e106386. Available from: https://pubmed.ncbi.nlm.nih.gov/25192280

3.	Ternes P, Feussner K, Werner S, Lerche J, Iven T, Heilmann I, et al. Disruption of the ceramide synthase LOH1 causes spontaneous cell death in Arabidopsis thaliana. New Phytol [Internet]. 2011 Dec 1;192(4):841–54. Available from: https://doi.org/10.1111/j.1469-8137.2011.03852.x

2) Abstract
• SPT: first mention of abbreviation without prior description

Response:

Thank you for pointing that out. This has now been amended.
Introduction:
• 26carbon: space missing
• Misspelling in sphingolipid: "sphinolipid biosynthetic pathway"
• Long chain bases (LCB) abbreviated twice (lines 67 and 153)

Response:

All errors have now been corrected. We appreciate the time taken to point them out.

3) Results:
• Text states that the model comprises 78 reactions, but S1 file enumerates 77

Response:

Thank you for bringing that to our attention. The 77 reactions in the S1 file do not include the reaction that was added to allow the model to capture orm activation. We accidently counted that reaction when writing the manuscript. That error has now been amended.
• Line 265: "(see Table 1 for details)”: should be Table 2

Response:

This has now been corrected.

4) Discussion:
• "Non-stationary isotope experiments will be conducted in future studies to determine the accuracy of this assumption and the results will be used to improve the model. "It is not clear who will perform these studies, but it would be good to rephrase it.
• Lines 382 – 387: same as above

Response:

Both cases have been addressed and are hopefully clear now.

5) Methods:
• 12 to15-day-old: space missing
• (5 d, 10 d, 15d and 20 d): space missing between 15

Response:

Thank you for pointing these out. They have been corrected.

• Line 441: it sounds like the sphingolipid components were measured in this study, although previously the authors mention that they were obtained from reference 16.

Response:

Thank you for pointing out this apparent contradiction. The sphingolipid components were measured in our lab but had already been published (in reference 16). We have clarified that in the edited manuscript.

• Line 474: please capitalize Gibbs to be consistent with the remainder of text
• Line 483: "This procedure is repeated thousands of times ": please add the number of repetitions
• If available, please provide the humidity for the growth of the Arabidopsis seedlings.

Response:

Thank you for pointing these out. Modifications were made for the first two points and the humidity has been provided (line 454).

6) Figures:
• Fig. 1 legend:
o What do the red flux values refer to?

Response:

The red value refers to the flux through class II ceramide synthase (CS II). This has been added to the figure caption.
o What is the unit of these fluxes, and where did they come from? The unit is mentioned in the S1 file.

Response:

The fluxes in figure 1 are unit-less as they were normalized by SPT. Please refer to line 209 and the subsection “Determining steady-state flux distributions” in the materials and methods section for detail.

• Fig. 2:
o The resolution could be improved
o The legend is insufficient to explain the figure

Response:

The resolution of Fig 2 has been increased. In addition, we have added a more detailed description in the figure caption as space limitations in the figure did not permit adding more detail to the legend.

• Fig. 3:
o The resolution could be improved. The dashed reference line is not visible.
o Legend: define ORM
o “A” & “B” missing

Response:

Thank you for pointing these out. The figure does not contain the acronym ORM. We believe the reviewer was referring LOH instead, which has now been defined.
7) Table 1:
• The reaction names are missing, it would be good to include them, at least in the caption.

Response:

The reaction names have been added to the table.
8) Table 2:
• Define overexpression (OE).
• Reference to the asterisk of concentration missing

Response:

Thank you for pointing these out. A definition of overexpression has been added. Furthermore, the asterisk was a typo that should not have been there and has been removed.
9) S1 file:
• What do the columns G & H refer to in the Excel spreadsheet?

Response:

Thank you for pointing that out. Those columns had been used for calculation purposes in a previous excel file and had inadvertently carried through. They have been omitted in the new submission.
• 77 reactions, but in the Results section 78 mentioned

Response:

Thank you. This has been addressed in response to minor edit #3.

10) S2 file:
• In the table, one of the ∆G'LB (kJ/mol) should be ∆G'UB (kJ/mol)

Response:

This error has been amended.

Reviewer #2:

1) A set of reference steady-state metabolite and enzyme concentrations are needed for integrating the kinetic models (as also described in lines 487 – 488). What values did the authors select? Or are the steady-state fluxes and concentrations computed robust to the reference level?

Response:

One of the main advantages of using the ensemble modeling framework is that it does not require measurement of metabolite and enzyme concentrations. This is because the computed concentrations are normalized by the reference steady-state concentrations. This is why solving the system of ODEs for the reference state results in a steady-state concentration of 1 ($\frac{x_{i,ss}}{x_{i,ss}^{ref}})$. Similarly, the computed enzyme concentrations are of enzyme complexes which have been normalized to the total enzyme concentration at the reference state. Therefore, metabolite concentration measurements are only needed when predictions of the actual (or absolute) concentration is desired. Furthermore, since the sampled kinetic parameters are also normalized by the reference steady-state metabolite and enzyme concentrations, measurements of both components are required to calculate the absolute values of these parameters. Since that was not the goal of this study, those measurements were not needed.

2) A set of 23 regulatory schemes on top of the base kinetic model were compared. Were these models trained or fitted with parameters in any ways (any regulatory interactions in a kinetic model should need parameters)? If additional parameters were introduced for each model (hypothesis), how were the parameters fitted?

Response:

We thank the reviewer for bringing up this point. It is correct that each regulatory interaction introduces a number of additional parameters (4 for inhibition and 8 for activation). The fitting of these parameters was done in a similar manner to the parameters associated with elementary steps in metabolic reactions as shown in the methods section and in reference 32 in the manuscript. We have also added a section to supplementary file 3 to demonstrate how the EM framework calculates these parameters. To clarify, parameters associated with regulatory interactions are not calculated after those associated with metabolic reactions, but rather both sets of parameters are calculated simultaneously and are treated in the same way. We hope that the additional section in supplementary file 3 (section ‘Determining kinetic parameters associated with regulatory reactions’) clarifies this concern.

3) Following up, if additional parameters were introduced, do they truly better capture the evaluation criteria or simply because more introduced parameters lead to better fit (potentially overfitting)? Do the model pass for example Akaike information criterion or some likelihood-ratio test (since these are nested models based on the base model) with the log-likelihood modeled by for example cross entropy for the classification evaluation criteria used? This will give more confidence to the identified hypotheses.

Response:

As the reviewer has pointed out, the issue of overfitting is always a concern when additional parameters are added into a model. The difficulty with using conventional methods, or the methods that the reviewers have kindly pointed to is that the EM approach doesn't exactly fit the parameters in the same way that a machine learning method would. In the EM approach, the parameters are not exactly fit, but rather sampled until values are found that result in the reference steady-state fluxes. This means that no matter how many parameters are used, the flux predictions for the reference-state will be the same. Therefore, metrics such as prediction error, specificity, and sensitivity can't be calculated.

Furthermore, after a perturbation, it is still not possible to quantify the mentioned performance metrics since the EM framework is only concerned with capturing the qualitative behavior resulting from each model. Hence, the question is not "which set of kinetic parameters performed better?" but rather "which set of kinetic parameters can recapture the qualitative changes observed in the perturbed state". It is for this reason that we, and previous works using this approach, are not able to quantitatively measure the effect of overfitting. However, what gives confidence to our identified observations is that several of the regulatory schemes that were tested had more parameters than the hypothesized scheme, some of which had the postulated scheme embedded in them. For example, scheme #7 (S3 file) which includes CS II activation by ORMs and ceramide inhibition of ORM (the proposed regulatory mechanisms) and has the additional regulation of CS I by ORMs. This scheme did not pass the implemented filtration steps. This observation entails that the hypothesized network did not pass the filtration steps due to overfitting. We have added an additional column with the number of parameters used in each scheme to the table in supplementary file 3.

4) Any additional interesting features by comparing the models that pass the filtering steps with the models that do not pass? This might generate new insights about how to engineer sphingolipid metabolism.

Response:

In our initial analysis, we had attempted to answer this question using multiple statistical and machine learning techniques including tests to compare the distributions of the kinetic parameters and clustering algorithms to determine whether the parameters from models that pass would form a separate cluster. However, our analysis did not reveal any interesting findings. Yet, being motivated by this comment, we went back to this question and looked at other ways in which we can perhaps gain further insights through this sort of comparison. We reasoned that looking at elementary kinetic parameters may not be sufficient simply due to the different combinations of parameter values that can result in the same apparent kinetics (i.e. kcat, Ks, and Km values). We therefore used the elementary kinetic parameters to calculate these apparent parameters (see Noor et al., 2013 or reference 49 in the paper). After doing this, we conducted a two-sample Kolmogorov-Smirnov test (KS test) to determine whether there was a statistical significance in the distributions of parameters that passed compared to those that did not. The test was conducted on each lumped parameter (e.g. comparing the distribution of kcat,1(passed) values to kcat,1(failed) values). A conservative cutoff of p<0.001 was selected to determine significance. Interestingly, we found that 3 out of the 250 lumped parameters displayed a significant difference between the two cases (passed vs. failed). Two of the three parameters were associated with LCB C4-hydroylase, the first branch-point in the sphingolipid pathway. This points to the essential role that SBH plays in branching di- and trihydroxy LCBs. Furthermore, the distribution of $k_{cat}^{+}$ values associated with the activated CSII pathway also displayed significant differences between the two subsets of models (passed vs. failed). This analysis indicates that LCB branching at SBH, and the binding of ORMs to CSII may play a vital role in maintaining homeostasis within the sphingolipid pathway.

We have added a paragraph to the results section (lines 330-346) and an additional supplementary file (S4 File) to convey these new findings. We are grateful to the reviewer for motivating this analysis.

5) Table 2 shows the evaluation criteria. How well does each of the regulatory models perform? Will be good to provide a detailed table or the matlab scripts to reproduce this.

Response:

The only regulatory scheme which consistently passes all of the applied filtration steps is the one shown in figure 4. We have added a supplementary table in Supplementary file 3 that lists all 23 schemes as well as the number of kinetic parameters associated with each scheme. The MATLAB script has also been provided in the github link below. The link has also been added to the manuscript (line 535).

<https://github.com/aalsiyabi/plant_sphingolipid_kinetic_model.git>

6) Also is the parameterized model made available?

Response:

The parametrized model and the steps required to reproduce it have now been uploaded to the github page associated with this project.

7) Line 432: that should be sum(|v|) instead of simply sum(v)?

Response:

Yes, thank you for pointing that out. We wrote all reactions so that they’re active in the forward direction, which is why we omitted the absolute sum. However, the general formulation does require the absolute sum to be present. We have amended this error.
